# Burn Wound Healing Activity of Hydroxyethylcellulose Gels with Different Water Extracts Obtained from Various Medicinal Plants in *Pseudomonas aeruginosa*-Infected Rabbits

**DOI:** 10.3390/ijms25168990

**Published:** 2024-08-18

**Authors:** Grigory Demyashkin, Tatiana Sataieva, Ludmila Shevkoplyas, Tatyana Kuevda, Maria Ahrameeva, Mikhail Parshenkov, Alexander Mimuni, Georgy Pimkin, Dmitrii Atiakshin, Vladimir Shchekin, Petr Shegay, Andrei Kaprin

**Affiliations:** 1 Department of Digital Oncomorphology, National Medical Research Centre of Radiology, 2nd Botkinsky Pass., 3, Moscow 125284, Russia; dr.shchekin@mail.ru (V.S.); dr.shegai@mail.ru (P.S.); kaprin@mail.ru (A.K.); 2 Laboratory of Histology and Immunohistochemistry, I.M. Sechenov First Moscow State Medical University (Sechenov University), Trubetskaya St., 8/2, Moscow 119048, Russia; misjakj@gmail.com (M.P.); a.mimuni@yandex.ru (A.M.); pim4im@yandex.ru (G.P.); 3 Department of Microbiology, Virology and Immunology, Crimean Federal University Named after V.I. Vernadsky, Order of the Red Banner of Labor Medical Institute Named after. S. I. Georgievsky, Lenina Blvd, 5/7, Simferopol 295006, Russia; tanzcool@mail.ru (T.S.); mila.shevckoplyas@yandex.ru (L.S.); 4 Department Field of Crop/Laboratory of Processing and Standardization of Essential oil Raw Materials, Research Institute of Agriculture of Crimea, Kievskaya St., 150, Simferopol 295043, Russia; green28t@yandex.ru (T.K.); kalimanova.1997@mail.ru (M.A.); 5 Research and Educational Resource Center for Immunophenotyping, Digital Spatial Profiling and Ultrastructural Analysis Innovative Technologies, Peoples’ Friendship University of Russia (RUDN University), Miklukho-Maklaya Str.6, Moscow 117198, Russia; atyakshin-da@rudn.ru; 6 Department of Urology and Operative Nephrology, Peoples’ Friendship University of Russia (RUDN University), Miklukho-Maklaya Str.6, Moscow 117198, Russia

**Keywords:** burn wound, essential oil water extracts, *Satureja montana* L., epidermis regeneration, anti-inflammatory action, cell proliferation

## Abstract

Burn injuries represent a significant problem in clinical practice due to the high risk of infection and the prolonged healing process. Recently, more attention has been given to natural remedies such as water extracts of various medicinal plants, which possess anti-inflammatory and wound healing properties. The aim of this study is to evaluate the efficacy and safety of *Satureja montana* L. and other water extracts in a burn wound model. The study involved male Californian rabbits (*n* = 52) divided into eight groups. Burn wounds were modeled on the animals and subsequently treated with gels based on *Satureja montana* L. and other water extracts. The reparative potential of the epidermis (assessed by Ki-67 expression), the state of local immunity (measured by the number of CD-45 cells), and the anti-inflammatory role of mast cells (measured by tryptase levels) were evaluated. Bacteriological and morphological studies were conducted. The most pronounced bactericidal, reparative, and immunostimulatory effects were observed after the treatment using a gel mixture of water extracts from *Satureja montana* L., *Salvia sclarea*, *Coriandrum sativum* L., and *Lavandula angustifolia* in equal proportions (1:1:1:1). The other gels also demonstrated high efficacy in treating burn wounds, especially when using a strain of *Pseudomonas aeruginosa* resistant to several antibiotics. Immunohistochemical studies showed a significant increase in the number of Ki-67-positive cells in the basal layer of the epidermis and a decrease in the number of CD-45-positive cells, indicating improved proliferative activity and reduced inflammation. This study confirms the hypothesis that the use of water extract mixtures significantly enhances the reparative potential, improves the immune response in the treatment of burns, and promotes wound healing. These findings pave the way for further research and the application of complex phytotherapeutic agents, specifically water extracts of medicinal plants containing phenols and antioxidants in burn wound therapy.

## 1. Introduction

Burn injuries pose a significant threat and are one of the causes of numerous fatal cases. The significant increase in the number of patients with skin defects in recent years has intensified the search, study, and introduction of new effective and safe wound-healing drugs into clinical practice [1]. Over the past decades, existing methods for treating purulent wounds have been repeatedly revised due to the growing incidence of purulent-inflammatory diseases and postoperative purulent complications. The modern range of surgical interventions (operations on abdominal and thoracic organs, bones and joints, vessels, etc.) creates a risk of postoperative wound suppuration, which often leads to a direct threat to the patient’s life. More than half of all deaths after surgery are associated with the development of nosocomial infectious and purulent complications.

Increasing bacterial resistance of hospital microflora to antibacterial drugs, an increased allergic background, and reduced body resistance make it difficult to treat patients with wound skin injuries. Many medications used in these cases have adverse effects on the epidermis [2,3]. The disadvantage of existing dosage forms for topical administration is the unidirectionality of action (for example, exclusively antimicrobial action), which is dangerous due to skin necrosis leading to the development of secondary wound infection of vital organs. In this regard, the creation of dosage forms of complex action (antiseptic, local anesthetic, and reparative) is relevant and is proposed in this study. The new dosage form must have complex antibacterial, antifungal, and regenerative effects and contain non-toxic low molecular components that ensure easy penetration into the wound surface area.

A sufficient number of medicinal plants have been recognized as valuable resources of antimicrobial compounds [1]. By now, the development and use of ready-made forms of medicinal plants produced by modern technology, despite their relevance, is not sufficiently covered in the available literature, and the accessible information remains contradictory.

One of the options for an effective solution to this problem is the use of natural substances. The therapeutic, antibacterial, wound healing, and anti-burn properties of many plants have long been known. This fact is reflected both in folk medicine and in many scientific studies and publications [4,5]. Indeed, herbal remedies are becoming increasingly popular in burn therapy due to their well-known antioxidant and anti-inflammatory properties [6]. These properties of essential oils and their derivatives have been thoroughly studied, but the reparative effects remain a subject of discussion.

In modern pharmacology, the use of water extracts obtained as the waste products during the production of essential oils of some medicinal plants is known, and these also have antibacterial and anti-inflammatory effects, etc. [7,8]. The technology of gel as a dosage form has been developed for many drugs for external use, but dosage forms for the treatment of wounds are mostly represented by ointments and creams. There are virtually no data on the technology of gels containing plant extracts, including those with several components.

Water extracts are intermediate products in the process of obtaining essential oil from a plant by steam or water distillation. During distillation, essential oils (EOs) are separated from soluble substances. Steam extracts more valuable substances from the plant than water, so the content of extracted substances in extracts is significantly higher than in a standard decoction. Water extracts have a few significant advantages, including their effectiveness under local exposure. Of particular interest are water extracts obtained from raw mountain savory (*Satureja montana* L.), which has strong antibacterial, anti-inflammatory, and wound healing properties. Aloe (*Áloë vera*), sage (*Salvia sclarea*), coriander (*Coriandrum sativum* L.), and lavender (*Lavandula angustifolia* are widely used in cosmetology and traditional medicine, and are known for their hemostatic, anti-inflammatory, antibacterial, analgesic, and regenerating properties. However, there are insufficient data in the specialized literature on the application of a mixture of various herbal components, despite their potential synergistic effects. Myrtle (*Myrtus communis* L.) contains a large number of biologically active components such as polyphenols, myrtucommulone, semimyrtucommulone, 1,8-cineole, α-pinene, myrtenyl acetate, limonene, linalool and α-terpinolene, which can help to reduce systemic antioxidant disbalance in burn disease. The combined use of water extracts from different plants may be more effective than their separate application, and this requires further research.

Mast cells (MCs) play an important role in the mechanisms of development of regenerative processes in the skin during a wound defect, which is associated with the release of pro- and anti-inflammatory mediators The morphofunctional characteristics of burn wounds of various degrees are still the subject of discussion among morphologists and surgeons; the mechanisms of proliferation and local immune response, especially the role of mast cell mediators, remain undisclosed. Another area is the evaluation of the properties of water extracts of *Satureja montana* L. and other medicinal plants on the burn wound model in rabbits.

## 2. Research Objective

The purpose of our study is the evaluation of the effects of gels containing water extracts of aloe (*Áloë vera*), savory (*Satureja montana* L.), sage (*Salvia sclarea*), coriander (*Coriandrum sativum* L.), and lavender (*Lavandula angustifolia*) in different combinations with the oral administration of myrtle leaf macerate (*Myrtus communis* L.) in the infected burn wound healing process in rabbits.

### Research Tasks

1. To evaluate the reparative potential of the epidermis based on the expression levels of the proliferation factor (Ki-67).
2. To assess the state of local immunity based on the number of CD-45 cells.
3. To establish the anti-inflammatory role of mast cells based on levels of tryptase distribution.

## 3. Results

Pressing a preheated copper plate produced skin damage typical to IIIa degree burn with the development of a white crust, which tended to make folds. Epidermal cells died but the basal lamina and deep skin appendages were partially preserved, and then the epithelium regenerated from these structures.

**Figure 1:**
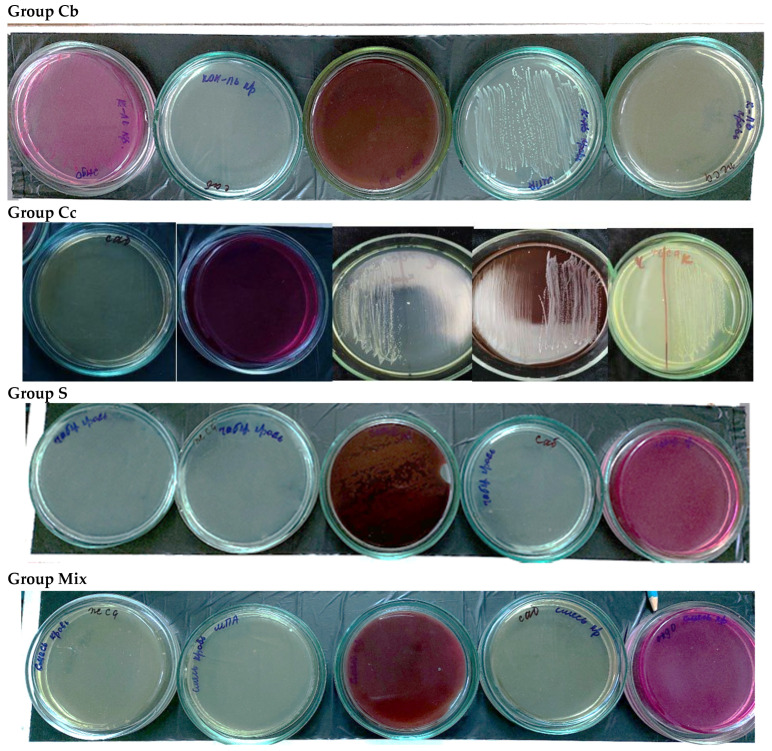
Bacteriological examination of blood samples. Culture on various media (from left to right: YSA, MPA, blood agar, Sabouraud, and Endo).

**Figure 2:**
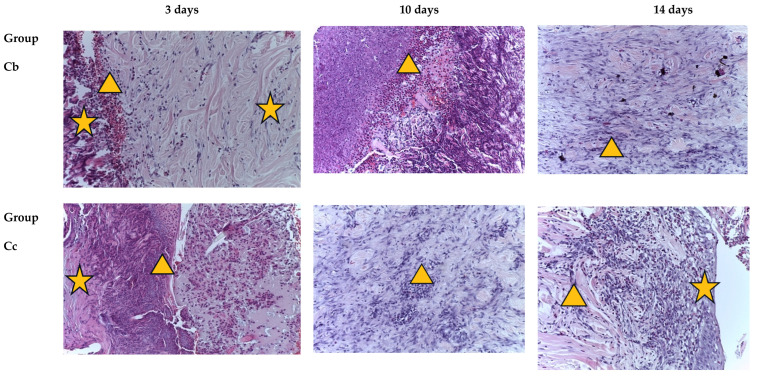

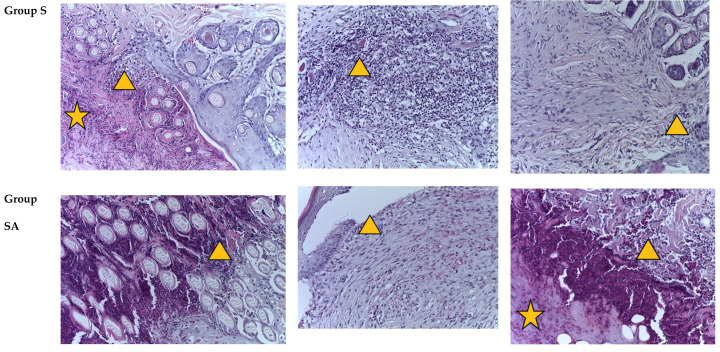

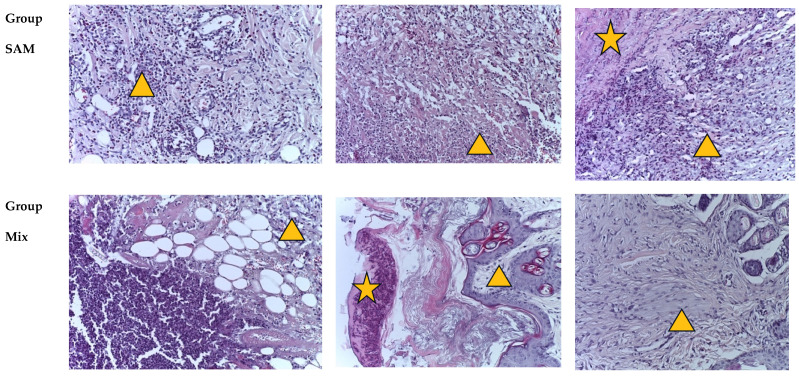
Burn wounds of the skin of the control and experimental groups on different days of the experiment. Histological examination. Staining with hematoxylin and eosin, magnification ×200. Symbols (figure captions): ★—detritus, ∆—inflammation.

A bacteriological examination of rabbit blood samples was conducted to identify secondary generalized infections (Table 1, Figure 1). The table illustrates the cultural characteristics of colonies and the type of culture growth on the Olkenitsky differential medium (iron–glucose–lactose agar with urea (IGLA)). Based on these characteristics, it can be concluded that *E. coli* was abundantly present in the blood of control animals, indicating a decreased immune status in this group. In contrast, no bacterial contamination was observed in the blood of animals treated with the gels containing water extracts. Figure 2 depicts the growth of *E. coli* from blood samples that were cultured on various media.

The bactericidal activity of blood serum (BABS), which is an integral indicator of the immune status of the body, was determined on the 21st day. At the same time, there were high BABS values in relation to staphylococcus and pseudomonas culture in rabbits of group VII, and the lowest values were in animals with a traditional method of treatment (group III).

### 3.1. Macroscopic Description

On the 7th day, a small amount of yellow–greenish viscous pus was observed in the burn wound area in animals of group III. The healing progress of burn wounds in animals of group III was similar to those treated with savory gel (group IV), but was less pronounced than in those treated with a mixture of water extracts (group VII). In the control group (Cb), the healing process was the slowest, and pus persisted throughout the experiment.

Some rabbits developed secondary staphylococcal infections in their ears, probably due to the burn stress. After the mechanical removal of pus, the ears were treated with savory, a mixture of water extracts, and levomecol ointment.

In dynamics, the rate of healing of a burn wound was the highest in those treated with a mixture of water extracts (group VII), second only to savory treatment (group SAM).

The hair growth of the skin after wound healing actively occurred after treatment with the water extract-containing gels, while the hair of the Cc group animals, on the contrary, grew very slowly.

### 3.2. Morphological Examination

The initial signs of recovery of the epidermis of a burn wound in group III (Cc) were observed on the 10th day. A similar pattern was observed in group V (SA). Also, on the 14th day, a weak inflammatory reaction was found in the papillary layer of the dermis, with a diffuse distribution of immunocompetent cells; the vascularization index was moderate (Figure 2).

In the burn wounds of animals of the VI group (SAM), the degree of reduction in cellular inflammatory infiltration was revealed starting from the 3rd day against the background of active collagen formation (an indirect sign of regeneration). At the same time, on the 14th day, cellular detritus and single neutrophils remained on the wound surface, and these persisted on the 21st day and occupied up to two-thirds of the area of the wound surface. At the same time, a pronounced vascular reaction was observed (Figure 2).

In the samples of the burn wounds of group VIII on the 7th day, cellular detritus was diffusely distributed in the epidermis, with signs of minimal damage, and a weak inflammatory reaction localized under the epithelium and in the papillary layer of the dermis. On the 10th day, thinning of the epidermis was detected, located mainly in the center of most micropreparations. Skin appendages, especially glands, were absent in some micro-preparations. The degree of inflammatory reactions gradually faded in dynamics; they were minimal on the 21st day (Figure 2).

An almost similar morphological pattern was observed in group IV (S), where signs of regeneration could be observed on the 7th day (Figure 2).

The maximum positive effect of treatment with water extracts was revealed in group VII (Mix). Already on the 3rd day, the demarcation zone was clearly visible. On the 7th day, cellular detritus was drained, and the epidermis was depleted. Single immunocompetent cells were located diffusely. On the 14th day, skin structures such as the epidermis and dermis were clearly visible, and corresponded to the structure of healthy skin (Ca groups) (Figure 2).

Thus, the stimulation of tissue repair of wound injuries using the proposed gels with plant extracts might be due to the increased synthetic activity of cells in the wound area, as evidenced by increased levels of nucleic acids (RNA, DNA). In wound sections, we noted increased basal activity of the epidermis, mostly expressed after application of the gel containing the mixture of water extracts of medicinal plants.

### 3.3. Immunohistochemical Examination

**Figure 3:**
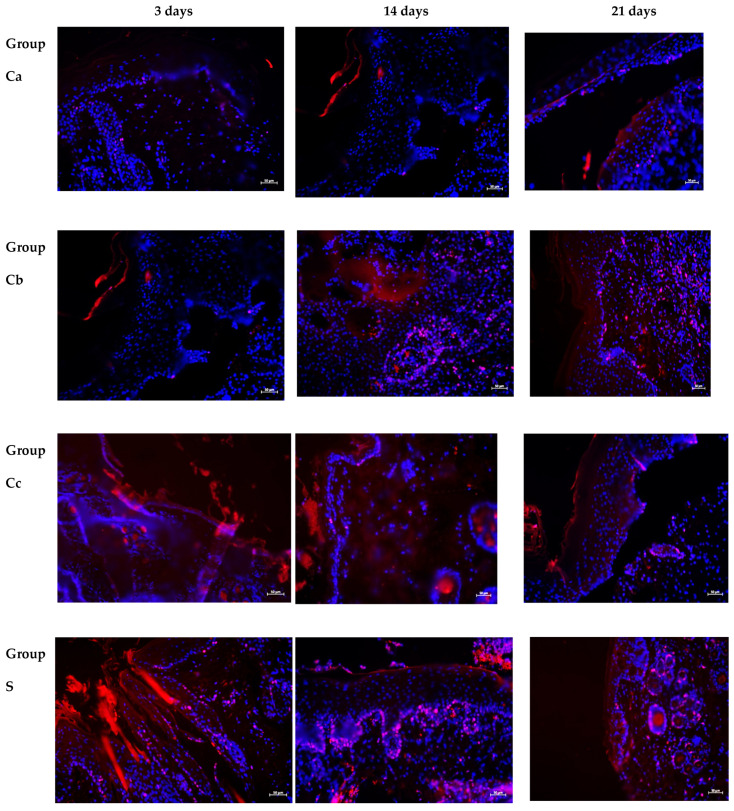

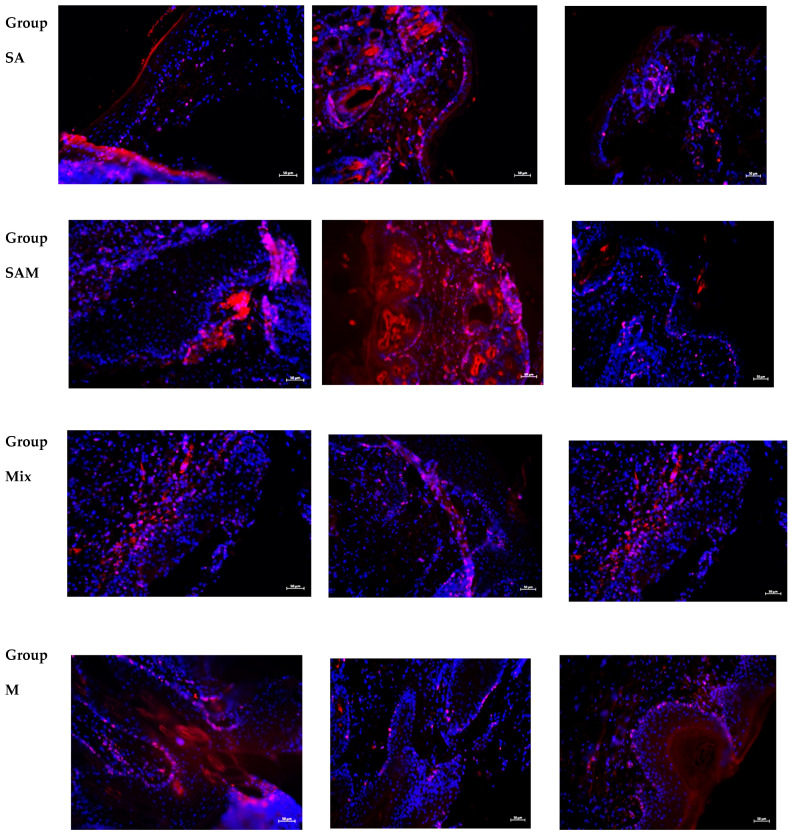
Burn wounds of the skin of the control and experimental groups at various study periods. Immunohistochemical study with antibodies to Ki-67 (red signal), nuclei—DAPI (blue signal); fluorescence microscopy; magnification ×400. Ki-67-positive cells were found in the epidermis (red signal).

**Figure 4:**
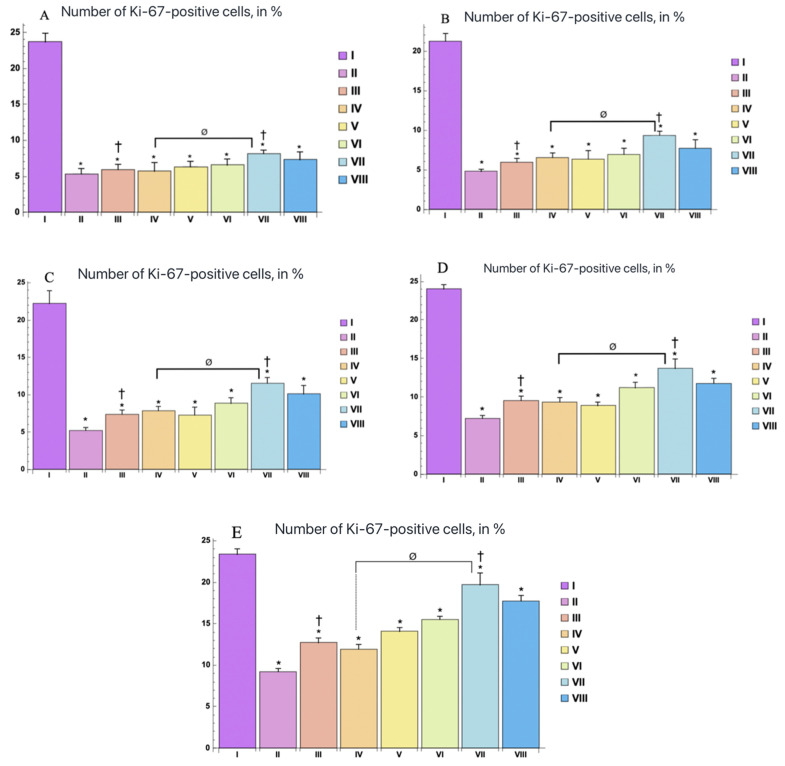
The number of Ki-67-positive cells in the epidermis of the control and experimental groups, (in %) at various study stages: (**A**) 3rd day, (**B**) 7th day, (**C**) 10th day, (**D**) 14th day, (**E**) 21st day. The experimental groups are numbered according to the design of the study. Statistically significant differences compared with the control group are indicated by * (*p* < 0.05); statistically significant differences between the III and VII groups are indicated by † (*p* < 0.01); statistically significant differences between the IV and VII groups are indicated by ø (*p* < 0.001).

During immunohistochemical examination, the nuclear staining for antibodies to Ki-67 was observed in keratinocytes of the basal layer of the epidermis and interfollicular epithelial cells, and no positive reaction was found in melanocytes and Langerhans cells. At the same time, the distribution of Ki-67-positive cells varied in groups and in different periods of the experiment (Figure 3 and Figure 4). The largest number of Ki-67-positive keratinocytes was found in the epidermis of the control group (Ca), and the smallest number in the II group (Cb), starting from the 3rd day, which persisted throughout the experiment.

At the same time, when assessing the distribution of expression of Ki-67-positively stained keratinocytes in the epidermis of burn wound samples during treatment with water extracts, the largest number of them was in group C, followed by an increase in dynamics (Figure 3 and Figure 4).

**Figure 5:**
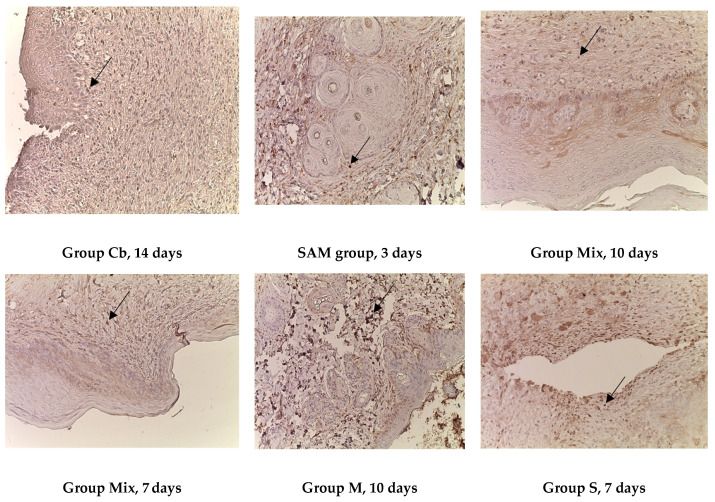
Burn wounds of the skin of the control and experimental groups on different days of the study. Immunohistochemical examination with CD-45 antibodies with hematoxylin staining of the nuclei; magnification ×400. CD-45-positive lymphocytes are found in the dermis (brown cytoplasmic staining, ↑).

**Figure 6:**
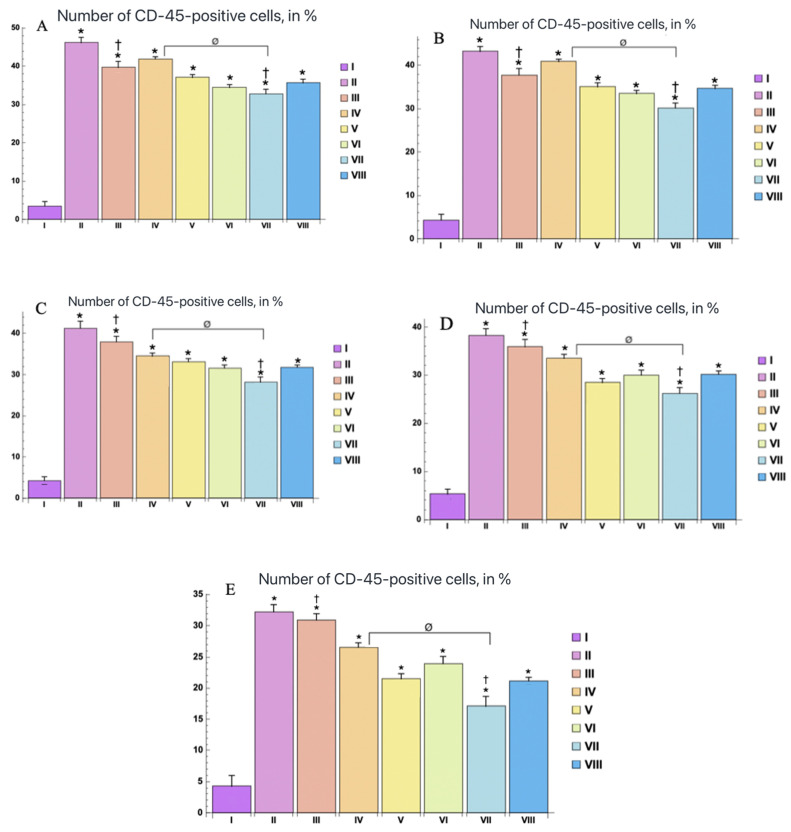
The number of CD-45-positive cells in the epidermis of the control and experimental groups (in %) at various study periods: (**A**) 3rd day, (**B**) 7th day, (**C**) 10th day, (**D**) 14th day, (**E**) 21st day. The experimental groups are numbered according to the design of the study. Statistically significant differences compared with the control group are indicated by * (*p* < 0.0001); statistically significant differences between III and VII are indicated by † (*p* < 0.001); statistically significant differences between IV and VII groups are indicated by ø (*p* < 0.0001).

Immunohistochemical quantification of CD-45-positive cells (a common marker of leukocytes) revealed a tendency for their numbers to decrease in groups C and M, starting from the 7th day (Figure 5 and Figure 6). On the contrary, the largest proportion of CD-45-stained leukocytes was found in groups II and III (Cb and Cc), and a small number of them was registered in the samples of the burn wounds of the VII and VIII groups (Mix and M) (Figure 5 and Figure 6).

During multiplex immunohistochemistry for the purpose of spatial phenotyping and multispectral visualization of the immune landscape of the tissue microenvironment in the burn wounds of animals in the control and experimental groups, the amount of tryptase was evaluated as a marker of identification of mast cells (MastCellTryptaseAntibody, Cy3) and correlation with proliferative activity (Ki-67).

**Figure 7:**
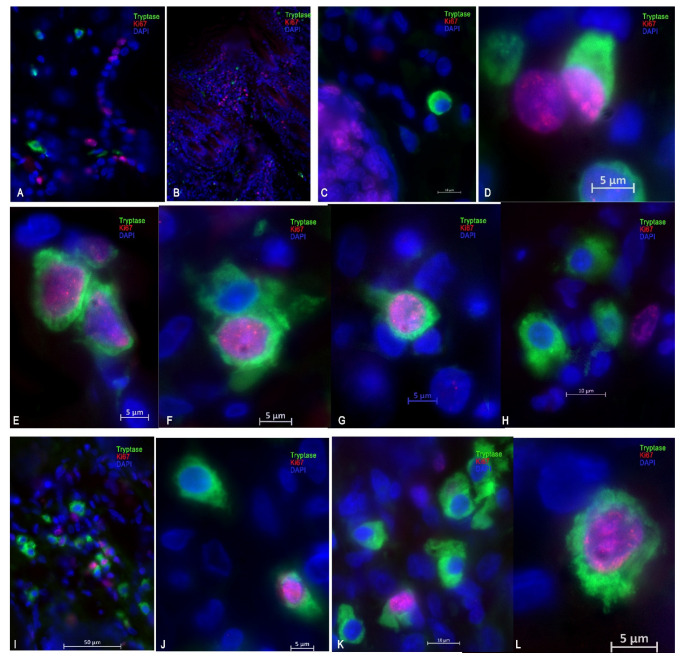
Mitotic activity of the mast cells in the experiment. (**A**) Cb group, 14th day; (**B**) Mix group, 21st day; (**C**,**D**) SA group, 21st day; (**E**) SA group, 3rd day; (**F**–**H**) SAM group, 3rd day; (**I**–**L**) M group, 3rd day.

The largest number of tryptase-positive mast cells in a burn wound was observed in the SAM group, starting from the 3rd day of the experiment, which persisted at days 10 and 21 against the background of increased proliferative activity (Table 2 and Table 3; Figure 7).

The number of MC in group VIII (M) increased at the beginning (on the 3rd day), and then decreased by the 10th day by almost 2.1 times. At the same time, proliferative activity was as high as possible during the first period of observation. On the 3rd day, co-expression of Ki-67 and tryptase was detected: co-localization of MCs with signs of mitotic division (Table 2 and Table 3; Figure 7).

In the SA group, the number of MCs increased throughout the entire duration of the experiment and reached maximum values by the 21st day. At the same time, the proliferative activity of tryptase-positive MCs increased (Table 2 and Table 3; Figure 7).

Thus, the largest amount of tryptase (mast cells) was found in animal samples of groups II and III (Cb and Cc), the average in groups S, SA, and SAM, and the minimum in groups VII and VIII (Mix and M). It should be noted that, in the remaining samples of burn wounds of the other groups, the number of MCs was approximately the same, and their lowest number was found in group C, and decreased gradually starting from the 10th day of the experiment. Consequently, cellular inflammatory infiltration gradually increased in the SAM group due to the possible biological effects of tryptase.

## 4. Discussion

Applied phytochemicals represented positive activity at different stages of the burn wound healing process by various mechanisms including antimicrobial, anti-inflammatory, and cell proliferative effects. Overall, the combination of several herbal extracts have shown rapid and marked activity in the management of burn wounds and therefore can be considered as an alternative source of treatment. 

Furthermore, various natural compounds with verified burn-induced wound healing potential can be considered as future natural drugs. Severe burn wounds need to be treated as soon as possible since any delay can postpone the healing process or result in infection. Although several topical preparations are present in the market for management of burn wounds, there is still an obvious lack of suitable drugs since most of the available products have antimicrobial activity rather than a wound healing effect, as well as their probable negative performance and toxicity. 

Medicinal plants such as *Satureja montana* L.), sage (*Salvia sclarea*), coriander (*Coriandrum sativum* L.), and lavender (*Lavandula angustifolia*) show good combination and can act together as wound-healing agents because of their vast variety of different constituents like alkaloids, essential oils, flavonoids, tannins, terpenoids, saponins, fatty acids, and phenolic compounds, which are potentially able to improve the healing process of burn wounds [9]. Rapid action, low cost, availability, and fewer side effects are other advantages of the selected herbal remedies. 

Many researchers all over the world are in the process of identifying and isolating the active components of medicinal plants responsible for their wound healing properties [1,4,6]. The safety of plants with wound healing properties should be also assessed because of their vast variety of phytochemicals potentially able to be toxic or allergic for damaged dermal cells. This is why the medicinal plants in our experiment were prepared in a topical form of steam water extracts, which significantly reduces the risk of toxicity and allergy. 

Skin burns can be initiated by thermal, chemical, radioactive, and other influences. Thermal burns are the most common and can lead to coagulation necrosis of tissues by causing functional and structural impairments. Without timely surgical interventions, a thermal burn can lead to serious complications, while the restoration of damaged areas requires prolonged treatment, along with the use of expensive medications, as well as long-term rehabilitation measures [9]. It is important to note that a burn with an area larger than half the skin can lead to multiple organ failure and death.

Skin burns may also occur among animals. For example, prolonged exposure to ultraviolet rays and other temperature influences can lead to burns of the deep layers of the skin, manifested by redness and necrosis [10]. Burn injuries of the skin can be caused by other causes, such as transdermal release of drugs during veterinary interventions. Some substances that promote the penetration of the active ingredient through the epidermis are known as sorption preparations. However, their use in veterinary practice, especially in high-absorption doses, can lead to skin damage [11].

Antibiotics are a common choice in skin burn therapy and also for prevention of secondary infection. However, in some cases they can cause allergic reactions that slow down the healing process. Another choice in the treatment of burn wounds is tissue self-sealing bandages, but their main problem is the high cost. Recently, pharmaceutical companies have been particularly interested in finding new effective and safe herbal remedies for the treatment and prevention of wound damage of the skin [12]. For the modern pharmaceutical industry, it is important to produce such substances that have anti-inflammatory, antioxidant, and other properties.

Unlike essential oils, the properties of which have been studied for many years and are widely described in the literature, there is much less information in the specialized literature about the properties of water extracts, obtained from plants after oil extraction. Previously, water extracts were considered to be a waste product in the production of essential oils by water–steam distillation. When water vapor passes through plant materials, they are saturated with the valuable, including water-soluble, components contained in the plants. Therefore, the chemical composition of water extracts is quite complex [13,14]. Nowadays, water extracts can also be obtained as an independent product, valuable for their cosmetic and medicinal properties. Unlike essential oils, they contain lower, but no less effective, concentrations of functionally active components, which allow them to be used in the treatment of wounds and burns in undiluted form [15,16]. If necessary, they can be easily diluted due to their water base, and, therefore, used as part of various dosage forms (irrigation solutions, gel dressings, ointments, etc.). Therefore, water extracts are considered a new product with mild but effective action, the comprehensive study of which is an important task [17,18]. Based on the literature review, the water extracts of identical plants contain almost the same substances as oils, only in a lower concentration, which allows us to apply them safely on mucous membranes and even open wounds without chemical injury, which might be caused by concentrated oils. However, there are practically no systematic studies aimed at directly comparing the properties of essential oils and the water extracts obtained from them.

Initially, our study was focused on water extract of *S. montana*, which is an essential oil known for its antifungal, antibacterial, antiviral, immuno-strengthening, analgesic, and tonic effects; it has been assigned class 5 toxicity—a non-toxic substance. It is also used in complex therapy in the treatment of combined bacterial and fungal infections, including COVID-19 [19]. The chemical composition of only the essential oil, which makes up 2.5–3.0% of the weight of the plant, has been well studied. In the essential oil of mountain savory (*S. montana*), phenols with high antibacterial activity make up 85.74%, including carvacrol—49.88, and thymol—0.23%; the remaining part is made up of phenol precursors—paracymene, gamma-terpinene, alpha-terpinene, and the terpene hydrocarbon alpha-pinene. The precursor of carvacrol, paracymene, has anti-inflammatory and antioxidant effects, and the terpinene group shows antimicrobial effects [20].

Due to the predominant phenolic component, the essential oil and water extracts of monarda (*Monarda fistulosa*) also have pronounced bactericidal, antiviral, fungicidal, anthelmintic, immunomodulatory, antioxidant, radioprotective, antisclerotic, cytotoxic, anti-inflammatory, and analgesic effects [21]. At the same time, *M. fistulosa* is considered to be one of the most powerful bactericidal agents of plant origin, capable of suppressing the growth of both gram–negative and gram-positive bacteria: *Klebsiella pneumoniae*, *Escherichia coli*, *Streptococcus pyogenes*, MRSA, *Streptococcus pneumoniae*, *Haemophilus influenzae,* and others [22]. At the same time, the essential oil of *M. fistulosa* has a stronger activity against pathogenic rather than beneficial microorganisms (*Bifidobacterium animalis* and *Lactobacillus casei*), i.e., it acts selectively [23]. Along with this, the formation of resistance towards essential oils is much slower than to antibiotics, and particular strains of Staphylococcus do not become resistant at all. *M. fistulosa* and phytoncidal plants in general can be successfully used in hospitals, schools, kindergartens, and other public places to reduce the concentration of staphylococci, streptococci, and other pathogenic microorganisms in the air. Fungicidal properties have been proven against Aspergillus fumigatus, Candida albicans, and Trichophyton mentagrophytes [24,25]. *Monarda fistulosa* is capable of inhibiting the growth of both filamentous and yeast forms of fungi of the genus Candida, including strains resistant to two-to-three antifungal drugs [26]. When studying the activity of 36 aromatic herbs in the ability to inhibit the growth of *T. mentagrophytes*, their volatile substances showed one of the best results [27].

The essential oil of coriander seeds (*Coriandrum sativum*) is considered a natural bactericidal agent, and the ingredient linalool has an antibacterial and antifungal effect [28]. *C. sativum* water extracts contain linalool—60–80%, geraniol—3–5%, cymol, geranyl acetate—up to 5%, and borneol—1–4%, and their acetic acid esters and aldehydes: n-decyl, decylene and isodecylene—0.2–2.5%; terpenes, pinenes, and fellandrenes. The disinfecting, detoxifying, antiseptic, antifungal, and antioxidant properties of coriander are suitable for eliminating skin diseases such as eczema, dryness, and fungal infections. Coriander leaf extract exhibits antioxidant activity and protects against photoaging of the skin caused by UV-B by regulating the expression of procollagen type I and MMP-1 [29].

Terpene alcohol linalool is found not only in *C. sativum* water extracts, but also in the water extracts of narrow-leaved lavender (*Lavandula angustifolia* Mill.) and clary sage (*Salvia sclarea*), and it has antiviral, antifungal, antibacterial, antiseptic, and decongestant effects [30].

The properties of the essential oil of clary sage (*Salvia sclarea*) cause a high content of esters—mainly linalyl acetate, linalool, and sclareol, a diterpene alcohol. Sclareol has anti-inflammatory activity. The mechanism of action of sclareol is associated with inhibition of nitric oxide production (suppression of inducible NO synthase of active macrophages), and cyclooxygenase-2 (COX-2), which leads to a decrease in the concentration of proinflammatory cytokines in the inflammatory focus. S. sclarea water extract is capable of providing antibacterial (a wide range of antibacterial activity, including gram-positive St. aureus, and gram-negative *E. coli*), fungicidal (antifungal, including *Candida albicans*), antiseptic, pronounced antispasmodic, and anti-inflammatory effects [31].

Essential oils of narrow-leaved lavender (*Lavandula angustifolia*) have depressive, sedative, anticonvulsant, antispasmodic, antioxidant, and antibacterial effects, and an influence on the degranulation of mast cells [32]. The main components of *L. angustifolia* essential oils are linalool, linalyl acetate, sesquiterpenes, flavonoids, and coumarins [33]. Water extracts of *L. angustifolia* have similar a composition to the main components; however, the main difference lies in the linalool content [34].

In the in vitro laboratory conditions, all the water extracts involved in the study showed a pronounced microbicidal effect, not only against pyogenic bacteria, but also against fungi, which was reflected in our publications [35,36]. An important aspect of this work was to reduce the level of bacterial contamination when using water extracts. This is confirmed by the absence of *E. coli* growth in blood cultures of animals treated with water extracts, unlike control groups. Among the animals of the Mix group, high values of bacterial activity of rabbit blood serum against staphylococcus and pseudomonas culture were noted, which is an integral indicator of immune status. The lowest level was recorded in animals of the II and III groups. The high bactericidal activity of water extracts is due to the presence of phenols and other active components, which, as shown in studies by other authors, have a powerful antimicrobial effect, as well as exhibiting antifungal activity, with the most sensitive microorganisms being *B. cereus* and *S. aureus*, and *P. aeruginosa* being the most stable [37].

Wound healing includes three phases [22]: I—inflammation phase; II—regeneration phase; and III—scar reorganization phase. Morphologically, three phases of cellular reactions are distinguished, depending on the predominance of individual cell types: leukocyte, macrophage, and fibroblastic [11,24,30]. These phases do not have sharp boundaries, rather a gradual transition from one to another is determined; they are characterized by the features of reactions on the part of all components of the cellular component.

As a result of macroscopic and histological studies, it was shown that the wound healing process in terms of degree and timing in animals with traditional levomecol treatment was almost identical to the monotherapy (S) group (group IV). At the same time, the best wound healing results were recorded in group VII, where the wound was treated with a gel composed of a mixture of water extracts of various plants in a ratio of 1:1:1:1. At the same time, healing was the slowest in the control group (Cb), and pus persisted throughout the experiment.

In other research, it was shown that bee honey from clover flowers, along with rosemary and chamomile oils, contributes to accelerating the healing of skin wounds in horses [38]. Other researchers expanded the composition of phytotherapeutic agents by adding sesame and olive oils [39,40]. The results showed that this new phytocomposition promotes skin regeneration, ensuring the healing of thermal burns within 28 days, while the epidermal layers became more mature compared with the groups treated with standard therapy drugs. These findings highlight the potential of phytogenic components in the treatment of burns, surpassing the effects of traditional drugs [41].

Immunohistochemical examination revealed that wound treatment with gels based on a mixture of savory water extracts (*Satureja montana* L.), sage (*Salvia sclarea*), coriander (*Coriandrum sativum* L.), and lavender (*Lavandula angustifolia*) in equal ratios of 1:1:1:1 significantly increases the number of Ki-67-positive cells in the basal layer of the epidermis relative to the standard therapy groups. This indicates increased proliferative activity, which confirms the data of previous studies on the stimulating effect of essential oils on cell growth and tissue regeneration [6]. The positive dynamics of skin regeneration was observed starting from the 7th day.

Analyzing the distribution of CD-45-positive cells, a decrease in their number was revealed in the groups treated with water extracts, starting from the 7th day, which indicates a decrease in the inflammatory response. This is consistent with the known anti-inflammatory properties of *Satureja montana* and other plants [42]. High values of CD-45-positive cells were recorded only in groups II and III.

In a multiplex immunohistochemical study, the control groups (Cb and Cc), where burns were treated without water extracts or just with boric acid and levomecol, showed the largest number of tryptase-positive MCs. On the 14th day of the experiment, high levels of cellular inflammatory infiltration persisted in the Cb group, indicating a pronounced inflammatory process and delayed wound healing. In the Cc group, where standard treatment was used, the number of MCs also remained high, which indicates ongoing inflammation and insufficient effectiveness of traditional therapy in reducing the inflammatory response. This is consistent with the data presented by the authors, where traditional antiseptics have shown limited effectiveness in reducing inflammation in the treatment of burns [43]. This confirms the need for research into alternative treatment methods, such as the use of essential oil water extracts. The use of a mixture of water extracts of mountain savory (*Satureja montana* L.) and aloe (*Áloë vera*) in the SA group showed a significant improvement in wound healing, confirming the data of El-Sherbeni SA [44], who also observed an acceleration of tissue regeneration when using extracts of other medicinal plants.

An increase in the proliferative activity of tryptase-positive mast cells (MCs) indicates a stimulating effect of phytocomponents on regeneration, which was also shown in the work of Al-Madhagy et al. [45], where plant extracts contributed to skin restoration. The most pronounced regenerative effect was observed in the SAM, Mix, and M groups. An increase in the number of MCs was accompanied by active collagen formation, which is an indirect sign of accelerated tissue regeneration. In addition, the SAM group showed a pronounced vascular reaction on the 21st day, indicating an improvement in microcirculation and tissue trophism in the burn area. In the Mix group, a minimum number of tryptase-positive MCs was noted, starting from the 10th day of the experiment, which indicates a decrease in the inflammatory response and an active healing process. In the M group, the number of MCs was high on the 3rd day, but by the 10th day the number had significantly decreased, indicating a rapid but short-term anti-inflammatory effect of *M. fistulosa*.

Mast cells play an important role in the mechanisms of development of regenerative processes in the skin in the case of a wound defect, which are associated with the release of pro- and anti-inflammatory mediators [46].

The literature reflects the issues of the morphofunctional state of MCs in different periods of the wound healing process, which can also contribute to the control of bacterial skin infections since activated MCs are crucial for the induction of protective innate immune responses to bacterial skin infections. Determination of the histoenzymological features of MCs in the skin made it possible to establish that they are activated at the very early stages of healing [46], as indicated by changes in the enzymatic activity of these cells during the degranulation actions of mast cells with other cellular components. Thus, the inflammatory phase is characterized by the transformation of monocytes into macrophages, with which MCs stimulate angiogenesis processes that determine the formation of granulation tissue. During the regenerative process, mobilization of all cellular components is observed, including mast cells, which provide a stimulating effect on the healing process [47].

There is an opinion that perivascularly located human MCs are capable of secreting collagen, which is associated with the participation of these cells in tissue remodeling processes [48]. It is most likely that the stimulating effect of mast cells on fibrosis processes is due not so much to the production of collagen by mast cells themselves, but to their activation of fibroblast function [49]. Mast cells play a key role in the formation of granulation tissue; the number of these cells increases during neoangiogenesis. Under physiological conditions, an increase in the number of these cells in the immediate vicinity of capillaries and lymphatic vessels has been noted. Immunohistochemically, the role of tryptase and chymase of mast cells in angiogenesis has been established. In particular, tryptase stimulates the proliferation of endothelial cells [50]. Mast cells determine the course of the regeneration phase, since the substances released during degranulation have a mitogenic effect on endotheliocytes and fibroblasts; histamine in certain doses stimulates the secretion of collagen by fibroblasts, which is a fundamental moment in healing.

There is an opinion that blocking the degranulation of MCs slows down wound healing and reduces the collagen content. Some authors believe that an increase of mast cells in the late stages is associated with the accumulation of biologically active substances. One cannot ignore the statement of researchers that the increase is associated with the maturation of young cells that do not participate in the reaction at the early stages of the wound healing process [51,52].

Mast cells are characterized by complex intercellular relationships that determine the direction and severity of various phases of the recovery process in the skin.

The use of water extracts of *S. montana* L. in various combinations with other plants contributes to a significant improvement in the healing of burn wounds. The reparative potential of the epidermis, estimated by the Ki-67 level, was significantly higher in the groups treated with water extracts, compared with the groups of classical therapy. This is due to an increase in the proliferative activity of keratinocytes and accelerated tissue repair.

The immune response was measured by the number of tryptase-positive mast cells (MCs) and CD-45-positive cells, and demonstrated a positive effect when using water extract-based gels. The revealed activation of collagen formation and improvement of microcirculation also indicates an improvement in regenerative potential. The most positive effect was achieved in the group receiving a mixture of water extracts (Mix), where the inflammatory response was reduced, and the restoration of the skin was faster than in the samples of other groups.

The wound healing stimulating effect of ointments with herbal extracts is explained mainly by the action of the phenolic substances contained in them, which can inhibit free radical oxidation of biomacromolecules, having an antibacterial effect, stimulating regenerative processes, limiting the severity of the inflammatory reaction, maintaining the liquid state of the blood, and other properties. The abundant phenolic compounds, as well as vitamins, polysaccharides, and macro- and microelements, provide a pronounced wound healing effect of the proposed gels with several water extracts, especially savory (*Satureja montana* L.), sage (*Salvia sclarea*), coriander (*Coriandrum sativum* L.), and lavender (*Lavandula angustifolia*). It is known that phenolic compounds perform a protective function, the most important element of which is the antioxidant effect [53]. They promote the restoration of tissue function and structure through active cell proliferation by limiting the intensity of free radical oxidation of biomacromolecules in wound healing processes and, also stabilizing tissue antioxidants [54]. However, oral administration of myrtle (*Myrtus communis* L.) did not significantly contribute to the local healing process. The obtained data on the stimulating reparative regeneration effect of our fully natural gels with the various herbal extracts are consistent with the results of other studies, which previously showed accelerated tissue regeneration under the influence of flavonoid-containing drugs and various tropical plant extracts. The activation of cell mitosis was observed after application of plant-based products containing phenolic compounds, vitamins, and macro- and microelements on wounds of the skin and diseases of the digestive organs. The stimulation of regeneration of the skin excisions in white rats after using an ointment with an extract of Amphimas pterocarpoides was definitely associated with the high content of phenolic compounds, which are also present in large amounts in the water extracts of *Satureja montana* and *Monarda fistulosa* of the Lamiaceae family, used in our experiment, which can be successfully grown in countries with a moderate climate [55].

Substances of a phenolic nature like carvacrol and thymol can inhibit increased peroxidation, limit intoxication, and ensure accelerated wound healing. Data on the pronounced wound healing effect of the obtained products are confirmed in the works of researchers who showed the dependence of wound healing on the intensity of free radical oxidation of biomacromolecules [56]. Moreover, some can put forward a hypothesis that free radical oxidation is a regulator of cell mitosis, and its activity can be associated with the limitation of cell reproduction and the slowing down of regenerative processes, and with the inhibition of this oxidation—the acceleration of reparative regeneration [57,58]. The data obtained by us are consistent with this point of view. The most pronounced wound healing effect of the water extract gels can be also explained by the fact that the bioavailability of microparticles of biologically active substances increases, and their uniform distribution in the gel base medium occurs, ensuring direct participation in the regulation of oxidation–reduction processes in the wound zone.

Thus, the use of water extract-based gels in the treatment of in vivo burn wounds significantly increases the reparative potential, improves the immune response in the treatment of burns, and promotes wound healing. These results open prospects for further research and the use of complex phytotherapeutic agents based on water extracts of medicinal plants for the treatment of various wound skin lesions. However, further research is needed to confirm these findings and expand the scope of application of water extracts. They will help to optimize treatment protocols and introduce new phytotherapeutic agents into clinical practice for more effective and safe treatment of wound skin lesions.

### Study Limitations

This study has some limitations that should be acknowledged when interpreting the results. Although animal models are a fundamental tool when studying human diseases, it is important to recognize differences between the species. Rabbit skin is characterized by a thin epidermis, which is composed of only one or two cell layers, displaying the thin stratum corneum and granular layer. In contrast, the proliferation index of rabbit skin is considered to be much higher than that of human skin. Compared with the skin of humans, rabbit skin is elastic and abundant, with a large surface of skin tissue relative to body size. In the rabbit, the healing process proceeds more slowly than in humans and it involves maximum wound contraction, prior to the initiation of cell migration and matrix remodeling. It is also well known that rabbits exhibit higher sensitivity to different skin irritants than humans.

## 5. Materials and Methods

**Figure 8:**
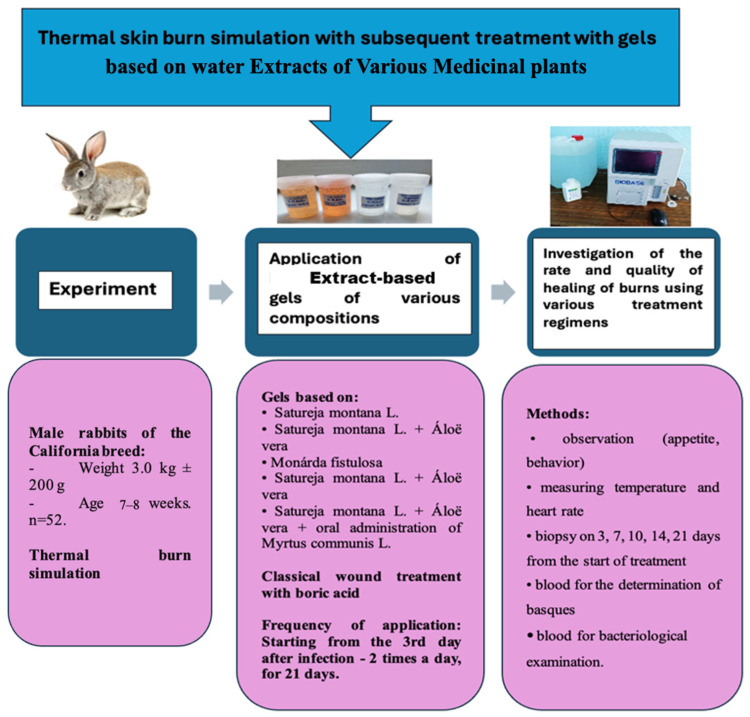
Design of the experiment.

Male rabbits of the California breed (3.0 kg ± 200 g; 7–8 weeks; *n* = 52) were divided into eight groups. The design of the study is presented in Table 4 and Figure 8.

**Figure 9:**
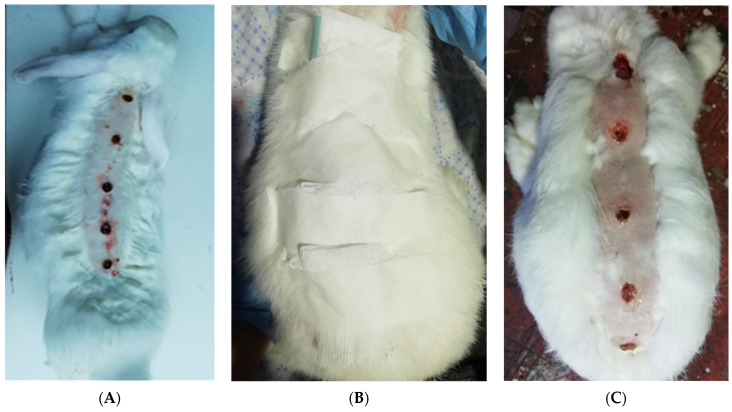
Modeling of thermal skin burn of the III-degree via macro photography. (**A**) Linear localization of burns of the interscapular area; (**B**) prevention of contamination of the wound by isolating it with a sterile patch; (**C**) the appearance of granulation tissue in the burn area.

Modeling of a thermal burn of the IIIa degree was performed on animals sedated by neuroleptanalgesia (“Zoletil^®^ 100”, Virbac, Paris, France; at a dose of 5 mg/100 g) by patching a 2 × 3 cm copper plate weighing 200 g preheated over the flame of an alcohol lamp to red-hot on the pre-shaved surface of the skin of the back of rabbits for 40 s with a pressure force of 2 Newtons on the tissue. In our model, to study the healing process on the same animal in dynamics, the 5 identical burns on the same animal were created in the direction from the interscapular region to the tail (Figure 9A).

In addition, 0.1 mL of a daily culture of *P. aeruginosa* (*Pseudomonas aeruginosa*) with a density of 1.0 units of turbidity according to McFarland was rubbed once in the wound surfaces 5 min after modeling of burns. The daily culture of *P. aeruginosa* was cultured in a concentration of 10^8–9^ CFU/mL on a liquid nutrient medium—meat-peptone broth. After the inoculation of microorganisms, the wound surface was closed with a sterile patch for 2 days to avoid additional external bacterial contamination (Figure 9B).

After modeling a thermal burn during wound treatment, the timing of scab formation, a decrease in edema of surrounding tissues, the onset of granulation tissue, the beginning of marginal epithelialization, and day of complete wound healing were noted, and the development of infectious complications was recorded (Figure 9C). During the entire period of the experiment, the behavior, appetite, and drinking habits of all animals were monitored, and body temperature was measured rectally by an electronic thermometer. The rate and quality of healing were evaluated in comparison with two control groups receiving classical treatment and in the absence of treatment.

The animals were placed in specialized cages (one animal per cage) and fed a commercial rabbit ration (Delta Feeds, Moscow, Russia); water was provided according to need throughout the study period. The acclimatization of the animals continued for one week, then the hair at the site of the thermal wound was removed using VeetTM cream (Veet, Cairo, Egypt) for two minutes.

The debridement and treatment of burn wounds in animals of all groups began on the 3rd day (the period for the development of infection) and was carried out 2 times a day. The duration of the experiment was 21 days. The animals were planned to be humanely sacrificed in case of “septic dermatitis” or complications that could not be treated. The selection of biomaterial for morphological examination was performed 3, 7, 10, 14, and 21 days after the corresponding burn wound. Wound cleansing was performed using surgical tweezers to hold necrotic tissue, a scalpel, and pointed scissors to cut off the scab from the underlying tissues. After opening the scabs and taking the biomaterial (fragments of damaged skin), the wounds were treated once with an aqueous solution of 0.5% chlorhexidine.

All manipulations were carried out in accordance with the “International Recommendations for Conducting Biomedical Research using Animals” (EEC, Strasbourg, 1985), the “European Convention for the Protection of Vertebrate Animals Used for Experiments or Other Scientific Purposes” (EEC, Strasbourg, 1986), and Guidelines for Conducting Biomedical Research on Care and the use of laboratory animals (ILAR, DELS). The study was approved by the Local Ethics Committee (Protocol No. 3 dated 10/21/2023).

### 5.1. Medicinal Plant Water Extracts and Gel Preparation

Water extract is a liquid, which is usually colorless or with a slight tint, with a pleasant aroma of the plant from which it is obtained by steam. The smell is not very pronounced, unlike the aroma of essential oil, since water extract is a low-concentration product, but it has almost the same properties as the corresponding essential oil. Since the concentration of active substances in water extracts is quite low, they can be safely used in a pure form, unlike essential oils. In addition, water extracts contain substances that do not remain during steam extraction in essential oils. These are water-soluble components of the plant that remain in water in the form of ions, and usually their percentage reaches 0.5%, but depending on the plant this figure may be higher. The yield of water extracts is on average from one to five liters per kilogram of the original plant material. Water extracts have an acidic pH of 3 to 6.2, which that can suppress the growth and activity of bacteria.

The applied gels from water extracts were obtained by mixing them with hydroxyethylcellulose at room temperature. Then the mixture was stirred and heated up to 40 °C; the hydroxyethylcellulose content was 2%. Water extracts of different plants were added in equal ratios.

The treatment was carried out topically by smearing the gels using sterile cotton buds, and conducted 2 times with an interval of 12 h between treatments daily.

*Bacteriological assay*. Rabbit blood was taken for bacteriological examination to determine the possible development of generalized infection. Blood samples (2–3 mL) were placed in sugar broth for growing for 24–48 h at a temperature of 37 °C. After that, streak culture was performed on solid diagnostic media to identify various microorganisms that could cause secondary infection (Yolk-salt agar (YSA), Endo and Saburo media, blood agar, and meat-peptone agar (MPA)). Petri dishes with cultures were incubated in a thermostat at 37 °C for 24–48 h. The identification of microorganisms was carried out using generally accepted methods for determining the cultural and biochemical properties of grown colonies, as well as by morphological signs of microorganisms in smears from colonies.

*Morphological study*. For a morphological assessment of the condition of the burn wound, fragments of the affected skin were fixed in a solution of buffered formalin, and then wiring (tissue histological wiring apparatus, Leica Biosystems, Hamburg, Germany) was embedded into paraffin blocks, from which serial sections (3 microns thick) were prepared, dewaxed, dehydrated, and stained with Mayer hematoxylin and eosin. Some sections were stained with Van Gieson to identify collagen fibers.

The degree of damage to the epidermis and the underlying dermis in micro-preparations were evaluated, and included the timing of the appearance of granulation tissue, as well as the structural maturity of the newly formed epithelium.

Special emphasis was placed on the etiology and intensity of the inflammatory reaction: the number of immunocompetent cells (including plasma cells), the degree of cell destruction in the focus of inflammation, edema, the growth activity of the epidermis, the direction of collagen fibers, fibroblasts, and the vascularization index.

### 5.2. Immunohistochemical Study

An immunohistochemical study was performed according to the standard protocol manually: sections were heated in a steamer with R-UNIVERSAL epitope recovery buffer (Aptum Biologics Ltd., Southampton, SO16 8AD, UK) at 95 °C × 30 min and quenched with endogenous peroxidase; primary antibodies were applied first, followed by secondary antibodies. Monoclonal antibodies to proliferation factor Ki-67 (ThermoFisher, Agawam, MA, USA; Clone MM1) and a common leukocyte marker, CD-45 (ThermoFisher, Agawam, MA, USA; Clone HI30), were used as primary for single immunohistochemistry, and for multiple immunofluorescence was used a mast cell marker—tryptase (ThermoFisher, Agawam, MA, USA; Clone TPSAB1/1961). For secondary antibody detection, the universal two-component HiDef Detection™ HRP Polymer system (“Cell Marque”, Lincoln, OR, USA), mouse/rabbit anti-IGG, horseradish peroxidase (HRP), and DAB substrate were used. Secondary antibodies (Dianova, Hamburg, Germany) for multiple immunofluorescences were conjugated to Alexa Fluor-488 (Thermo Fisher Scientific, Eugene, OR, USA). The final concentration of secondary antibodies was 5 to 10 μg/mL PBS. Single and multiple immunofluorescence labeling were performed according to standard protocols. A combined protocol including standard tryptase immunohistochemistry was used to simultaneously detect tryptase-positive MCs and Ki-67. Cell nuclei were stained with Mayer’s hematoxylin. In multiplex immunohistochemistry, nuclei were detected using DAPI (#D9542-5MG; Sigma, Steinheim, Germany).

Histological and immunohistochemical analyses were performed using a video microscopy system (Leica DM3000 microscope, Hamburg, Germany; DFC450 C camera (Leica Microsystems, Wetzlar, Germany), Platrun LG computer (LG, Seoul, Republic of Korea) and Leica Application Suite (LAS) Version 4.9.0 image processing and analysis software. The number of immunopositive cells was counted in 10 randomly selected fields of view at ×400 magnification (in %). Post-multiplex slides were viewed on a ZEISS Axio Imager.Z2 equipped with a Zeiss alpha Plan-Apochromat (Carl Zeiss AG, Oberkochen, Germany) 100×/1 46 Oil DIC M27 objective lens, a Zeiss Objective Plan-Apochromat 150×/1.35 Glyc DIC Corr M27 objective lens, and a ZEISS Axiocam 712 colour digital microscope camera. Images were processed using the Zen 3.0 Light Microscopy Software Package”, “ZEN Module Bundle Intellesis & Analysis for Cells 2022, 11, x Light Microscopy”, and “ZEN Module Z Stack Hardware” (Carl Zeiss Vision, Jena, Germany). The proportion of Ki-67-positive cells in the epidermis per unit area and their absolute number of Ki-67-positive cell counts were determined using the open-source digital image analysis software QuPath (Ver. 0.5.0). Also, mast cell (tryptase+) counts in the dermis of the skin were performed using the open-source digital image analysis software QuPath. 

*Statistical analysis*. All statistical analyses were performed using the computer program SPSS 12.0 for Windows (IBM Analytics, New York, NY, USA). All data are presented in the format of the mean value ± standard deviation (M ± SD). The Kolmogorov–Smirnov test was used for each sample separately to test the hypothesis of the normality of the distribution of values. In the case of a normal distribution, the Student’s *t*-test was used. The differences between the samples were considered statistically significant at a significance level of *p* < 0.05, established before the start of the analysis.

## 6. Conclusions

Morphological aspects of the participation of various cells in the restoration of tissue integrity during the wound healing process continue to be the focus of attention, since the degree of involvement of the cellular component in this process has great importance for determining structural changes during healing, and the study of healing mechanisms for the purpose of managing this process is one of the most important problems of theoretical and clinical medicine.

The present study confirms the hypothesis that the use of medicinal plant water extracts in gel mixtures significantly enhances the reparative potential, improves the immune response, and promotes the healing of burn wounds. The most pronounced bactericidal, healing, and immunostimulatory effects were achieved with the use of a mixture of water extracts. The herbal gels have demonstrated high efficacy in treating burn wounds, especially when dealing with strains of *Pseudomonas aeruginosa* resistant to multiple antibiotics. The results of this study indicate the future potential for using herbal water extracts in hospital settings for treating burns of varying severity and preventing hospital-acquired infections in the burn wounds. 

## Figures and Tables

**Table 1:** Growth and cultural characteristics of colonies grown from blood cultures of control and experimental groups of animals.

Culture Medium’s Name	Group Cb	Group S	Group SA	Group SAM	Group Mix	Group M
**MPA**	Small, translucent colonies, R-form, G-rods subcultured on the IGLA correspond to *E. coli* growth type, *oxidase-*.	Lack of growth	Lack of growth	Insignificant amount of small, translucent colonies, R-form	Lack of growth	Lack of growth
**Blood agar**	Greenish–grayish small colonies with an uneven edge on IGLA and endo growth correspond to *E. coli*	Lack of growth	Lack of growth	Lack of growth	Lack of growth	Lack of growth
**Endo**	Small colonies with a metallic sheen on the IGLA, growth is typical for *E. coli*	Lack of growth	Lack of growth	Lack of growth	Lack of growth	Lack of growth
**YSA**	Lack of growth	Lack of growth	Lack of growth	Lack of growth	Lack of growth	Lack of growth
** Sabouraud **	Lack of growth	Lack of growth	Lack of growth	Lack of growth	Lack of growth	Lack of growth

**Table 2:** Relative amount of tryptase-positive MC in the skin (in %, relative to other dermal cells).

Experimental Group	Period of the Experiment, Day				
**P**	3	7	10	14	21
**SA**	-	-	2.2	4.2	7.2
**S**	-	2.7	3,4	-	3.3
**SAM**	8.6	-	-	13.1	12.4
**Mix**	-	-	3.4	2.4	1.2
**M**	9.2	4.5	4.2	-	-
**Cb**	4.2	-	3.4	3.5	-

**Table 3:** Proliferative activity of tryptase-positive MCs in the skin (relative, in %).

Experimental Group	Period of the Experiment, Day				
	3	7	10	14	21
**SA**	-	-	3.7	2.4	6.8
**S**	-	0.5	1.4	-	1.7
**SAM**	5.4	-	-	5.3	2.1
**Mix**	-	-	0.3	0.4	0.6
**M**	4.2	1.4	3.8	-	-
**Cb**	0.5	-	2.3	3.2	-

**Table 4:** Research groups.

No.	Group, Name	Quantity, *n*	Manipulations Performed
I	Control group 1 (Ca)	10	An intact group of animals that have not been burned and are in the same conditions as experimental animals.
II	Control group 2 (Cb)	6	A group of animals that received a thermal skin burn of the IIIa degree without treatment.
III	Control group 3 (Cc)	6	A group of animals with skin burns of the IIIa degree, followed by traditional treatment of the wound with boric acid and applying a napkin soaked with betadine with levomecol.
IV	Experimental group (S)	6	A group of animals with a IIIa burn followed by topical wound treatment with a gel based on mountain savory water extract (*Satureja montana* L.).
V	Experimental group (SA)	6	A group of animals with a IIIa skin burn followed by wound treatment with the gel based on mountain savory (*Satureja montana* L.) and aloe (*Áloë vera*) water extracts in a ratio of 1:1.
VI	Experimental group (SAM)	6	A group of animals with grade IIIa skin burn followed by wound treatment with the gel based on mountain savory (*Saturej amontana* L.) and aloe (*Áloë vera*) water extracts in a ratio of 1:1, and oral administration of aqueous myrtle leaf macerate (*Myrtus communis* L.) in a dilution of 1:100 (2 mL of water extract per 200 mL of water).
VII	Experimental group (Mix)	6	A group of animals with a IIIa burn followed by wound treatment with the gel made from a mixture of water extracts of savory (*Satureja montana* L.), sage (*Salvia sclarea*), coriander (*Coriandrum sativum* L.), and lavender (*Lavandula angustifolia*) in equal proportions of 1:1:1:1.
VIII	Experimental group (M)	6	A group of animals with a grade IIIa skin burn followed by wound treatment with the gel based on mountain savory water extract (*Satureja montana* L.) and monarda (*Monarda fistulosa*) in a ratio of 1:1.

## Data Availability

The study did not generate publicly available archival data.
